# Hand Book of General Therapeutics

**Published:** 1887-01

**Authors:** 


HANDBOOK OF GENERAL THERAPEUTICS. By Prof. H. Von Ziemssen; in seven volumes.

We have received the fifth volume of this useful work. For full description of the work, its aim and scope, the reader is referred to Vol. I, Nos. 2 and 6, of this Journal. It is being published uniform, in cloth, hy the celebrated house of Wm. Wood & Co., and will be finished in two more volumes. No physician who expects to keep fully up with the science of medicine, should fail to acquaint himself with these works, which contain the most modern views as to therapeutics of all known diseases.

